# The Diagnostic profile and clinical course of patients with rheumatic diseases in the medical intensive care unit

**DOI:** 10.55730/1300-0144.5673

**Published:** 2023-09-06

**Authors:** Kaniye AYDIN, İpek TÜRK

**Affiliations:** 1 Division of Medical Intensive Care Unit, Department of Internal Medicine, Faculty of Medicine, Çukurova University, Adana, Turkiye; 2 Division of Rheumatology, Department of Internal Medicine, Faculty of Medicine, Çukurova University, Adana, Turkiye

**Keywords:** Flare-up, infection, intensive care unit, mortality, systemic lupus erythematosus, systemic rheumatic disease, platelet to lymphocyte ratio

## Abstract

**Background/aim:**

Immunosuppressive and immunomodulatory treatments developed in recent years as a result of a better understanding of the pathophysiology of systemic rheumatic diseases (SRDs) improve the prognosis. Despite medical advances, individuals with SRDs at any stage may require intensive care and have a high mortality rate. The aim of this study was to investigate the demographic and clinical characteristics of patients with rheumatic diseases admitted to the intensive care unit (ICU), and the factors associated with the risk of mortality.

**Materials and methods:**

This was a retrospective, cross-sectional study that included patients with rheumatic diseases in the medical ICU. Factors of ICU 28-day mortality were identified by multiple-variable logistic analysis.

**Results:**

A total of 127 patients with SRDs admitted to the medical ICU were enrolled. Systemic lupus erythematosus (SLE) (32.3%) was the most common diagnosis of SRDs in patients admitted to the ICU. The reasons for admission to the ICU were combined infection and primary SRD flare-up (35.4%), primary SRD flare-up (22%), SRD-unrelated reasons (22%), infection (17.3%), drug side effects (3.9%), and SRD-related complications (0.8%). The most common organ dysfunctions before (49.6%) and during (77.2%) admission to ICU were in the respiratory system. The 28-day mortality was 78 (61.4%). While the maximum procalcitonin, serum lactate, and blood urea nitrogen (BUN) levels were higher in the nonsurvivor group, the platelet and serum albumin levels were statistically significantly lower than those in the survivor group (p < 0.05). Acute respiratory failure (ARF), the presence of septic shock, the need for invasive mechanical ventilation (IMV), BUN level, and low platelet-lymphocyte ratio (PLR) were significant in the final multiple-variable model.

**Conclusion:**

Significant predictors of mortality in patients with rheumatic diseases may include ARF, septic shock, the need for IMV, and high BUN and low PLR levels.

## 1. Introduction

Systemic rheumatic diseases (SRDs) are accompanied by inflammation, organ involvement, and autoimmunity. They cause significant morbidity and mortality if not recognized and treated. As the pathophysiology of SRDs begins to be understood, a response to treatment with immunosuppressive and immunomodulatory agents can be obtained and remission can be achieved. However, the desired response cannot be obtained in every patient, complications such as susceptibility to infection due to immunosuppression, disease flare-up, and drug side effects are encountered. While some patients are diagnosed with SRDs at the time of admission to the ICU, some individuals require admission for the above-mentioned reasons. One-third of these patients require admission to the intensive care unit (ICU), which accounts for approximately 1.4% of the patients admitted to the ICU [1, 2]. In the literature, the most common rheumatic diseases requiring ICU were systemic lupus erythematosus (SLE) and rheumatoid arthritis (RA) [3, 4]. Considering the reasons for admission to the ICU in the literature, the most common were disease flare-up, infection, or a combination of both [1, 2, 5].

Knowledge of the clinical features, outcomes, and mortality-related factors of patients with rheumatic disease hospitalized in the ICU will contribute positively to the management of these patients. The aim of this study was to investigate the type of disease, etiology, and demographic and clinical characteristics of patients with rheumatic diseases admitted to the ICU. Additionally, it was planned to examine the factors associated with the risk of mortality in these patients.

## 2. Materials and methods

### 2.1. Patient population

This retrospective study was conducted in the medical ICU between January 1st, 2010, and June 15th, 2022. Inclusion criteria were patients ≥18 years who had SRDs. Patients with multiple ICU admissions were included in the study once, and only their data at the time of initial ICU admission were analyzed. Exclusion criteria were patients <18 years, and pregnant women. The local ethics committee approved the study protocol (Date: 22/07/2022, Reference number: 18/124). Between 2010 and 2022, a total of 6826 patients hospitalized in the medical ICU were screened for the presence of SRDs. SRDs were detected in 127 patients. The post hoc power analysis of the study was tested using the G Power program based on the mean differences between the groups (78 vs. 49 patients). The analysis was single-ended. With an effect size of 0.5 and a margin of error of 0.05, a total sample of 127 patients reached a power of 0.86. The results suggested that the sample was of sufficient size.

### 2.2. Clinical information about the patients

Demographic characteristics (age, gender), type of SRD, duration of diagnosis, Charlson comorbidity index, acute physiology and chronic health evaluation (APACHE) II score, laboratory parameters on ICU admission (complete blood count, C-reactive protein (CRP), procalcitonin, blood urea nitrogen (BUN), creatinine, glomerular filtration rate, serum albumin, alanine aminotransferase, ferritin, serum lactate), and laboratory parameters during ICU stay (maximum CRP and procalcitonin levels) were recorded from the hospital database. During the data collection period, the diagnosis of SRDs was determined using standard criteria for these conditions [6–14]. The CRP-albumin ratio, neutrophil-lymphocyte ratio (NLR), platelet-lymphocyte ratio (PLR), and lymphocyte-monocyte ratio were also determined. The locations where the patients were admitted to the ICU were divided into 3 categories: emergency room, inpatient service, and other ICU. In the ICU, the current results of the patients were evaluated with the relevant departments, and their reasons for hospitalization were classified.

The reasons for ICU admission were divided into 6 categories: infection, primary SRD flare-up, combined infection and SRD flare-up, SRD-unrelated complications, drug side effects, and SRD-related complications. Patients with fever were first investigated for the presence of infection. The presence of symptoms, a physical examination, and laboratory (e.g., complete blood count, urinalysis, urine sediment, CRP, procalcitonin, culture, serology tests) and imaging findings suggestive of infection supported the diagnosis of infection. Culture, serology, or polymerase chain reaction positivity and a low activation score confirmed the diagnosis of infection. For the diagnosis of sepsis and septic shock, criteria from the 2001 International Sepsis Definitions Conference were used for patients hospitalized in the ICU between 2010 and February 2016, and the Sepsis 3 criteria were used for patients hospitalized after February 2016 [15, 16]. The diagnosis of a primary SRD flare-up was determined by evaluating the disease activation criteria. Patients with hematological abnormalities were investigated for SRD involvement, other comorbidities, or treatment-related causes. Initially, detailed medical and medication histories were obtained, and a physical examination (e.g., splenomegaly, lymphadenomegaly) was conducted for all of the patients. Peripheral blood smears from all of the cytopenic patients were analyzed. Iron parameters, vitamin B_12_, folic acid, lactate dehydrogenase levels, reticulocytes, Coombs tests, coagulation parameters, fibrinogen levels, liver function tests, and hepatitis markers were assessed if indicated. If sepsis-related cytopenia was suspected, further evaluation was performed if the cytopenia did not improve after sepsis treatment. Imaging was performed in patients deemed necessary for organomegaly or cirrhosis. If the patient had unexplained cytopenia, a bone marrow aspiration and biopsy were performed. After all of the investigations had been carried out, the cause of the hematological abnormality was determined in consultation with the relevant departments. The admission of the patient to the ICU because the organ involvement resulted in complications in another system was defined as an SRD-related complication. Drugs used were analyzed and evaluated in terms of side effects. First, the patients were examined in terms of organic causes and disease activation. After they were ruled out, if the side effect improved after the drug was discontinued, it was accepted as a drug complication. Reasons for admission to the ICU, such as trauma or the need for the ICU in the postoperative period, that were not related to the autoimmune process were defined as SRD-unrelated reasons.

Organ involvement that existed prior to hospitalization and organ involvement that resulted in hospitalization in the ICU were classified separately as respiratory, kidney, neurological, cardiovascular, gastrointestinal, hematological system, and other [17]. Acute kidney injury (AKI) was diagnosed according to Kidney Disease: Improving Global Outcomes (KDIGO) 2012 criteria [18]. First, renal ultrasonography was performed in terms of postrenal events. Urine sediment was analyzed for renal involvement, and the patients were evaluated for proteinuria.

The treatments of the patients who received immunosuppressive therapy in the last 3 months were recorded. The steroid dose was recorded on the study form and classified as <7.5, 7.5–30, 30–100, and >100 mg.

The presence of infection, sepsis, and septic shock; presence of AKI, presence of acute respiratory failure (ARF); treatments applied in the ICU (inotropic and vasopressor therapy, noninvasive mechanical ventilation (NIMV), IMV, renal replacement therapy (RRT), and therapeutic apheresis); and the length of stay in the ICU (days) were evaluated retrospectively.

The outcomes of the patients were investigated as death in the ICU, 28-day mortality, recurrent hospitalization in the ICU in the last 6 months, and 6-month mortality.

### 2.3. Statistical analysis

The Kolmogorov-Smirnov test was used to confirm the normality of the distribution for continuous variables, which were presented as the mean ± standard deviation (SD) for normally distributed data, and median (Q1–Q3) for not normally distributed data. The categorical variables were presented as the number of categories (percentage). According to 28-day mortality, the patients were divided into 2 groups, as survivors and nonsurvivors. The student t test or Mann-Whitney U test was used to compare continuous variables, while the chi square test or Fisher exact test was used to compare categorical variables. The relationship between the variables was assessed using the Pearson or Spearman test. Logistic regression models were used to identify associations between 28-day mortality and the other variables. Factors included in the multivariate regression model were selected for their clinical relevance among variables yielding p < 0.25 in the univariate analyses. The final multiple-variable model was selected via a forward-looking stepwise procedure. IBM SPSS Statistics for Windows 20.0 (IBM Corp., Armonk, NY, USA) was used for all of the analyses. For all tests, p < 0.05 was considered statistically significant.

## 3. Results

Between 2010 and 2022, a total of 6826 patients hospitalized in the medical ICU were screened for SRDs. The study involved 127 patients, 83 (65.4%) of whom were female. The mean age of the patients was 48.55 ± 17.05 years. SLE (32.3%) was the most common diagnosis of SRDs in patients admitted to the ICU, followed by RA (17.3%) and antineutrophil cytoplasmic antibody (ANCA)-associated vasculitis (AAV) (17.3%). The reasons for admission to the ICU were combined infection and primary SRD flare-up (35.4%), primary SRD flare-up (22%), SRD-unrelated reasons (22%), infection (17.3%), drug side effects (3.9%), and SRD-related complications (0.8%). A SLE patient with newly developing renal involvement was admitted to the ICU because of sepsis and convulsions due to hyponatremia. The reasons for admission of this patient were considered a combined infection and SRD flare-up and an SRD-related complication.

The respiratory system (49.6%) was the most commonly involved organ before patients were admitted to the ICU, followed by renal (40.2%) and hematologic (12.6%) involvement. The most common organ dysfunction leading to hospitalization was also the respiratory system (77.2%). When examining whether immunosuppressive agents were used in the last 3 months, the data of 125 patients were obtained, which showed that 64 (50.4%) used immunosuppressive agents. Of the patients, 84.3% had sepsis and ARF. Shock was present in 78 patients (61.4%). When considering the causes of shock, septic shock was the most common (57.5%), followed by cardiogenic (15.7%) and hypovolemic (1.6%) shock. NIMV was required in 56 (44.1%) patients, IMV in 85 (66.9%), RRT in 44 (34.6%), vasopressor/inotropic therapy in 81 (63.8%), and therapeutic apheresis in 37 (29.1%).

Table 1 summarizes the demographic and clinical characteristics of the patients according to 28-day mortality. In the nonsurvivor group, the presence of ARF, shock, sepsis, septic shock, cardiogenic shock, AKI, and pneumonia, the need for IMV, and inotropic/vasopressor therapy were statistically substantially higher than in the survivor group (p < 0.05).

Table 2 shows the laboratory parameters of patients with SRDs admitted to the ICU according to 28-day mortality. While the procalcitonin maximum, serum lactate, and BUN levels were greater in the nonsurvivor group, platelet and serum albumin levels were statistically significantly lower than those in the survivor group (p < 0.05).

The ICU, 28-day, and 6-month mortality rates of 127 patients were 63 (49.6%), 78 (61.4%), and 83 (65.4%), respectively. In terms of recurrent admission to the ICU, 22 (17.3%) patients were hospitalized once, 5 (3.9%) were hospitalized twice, and 1 (0.8%) was hospitalized 3 more times.

Table 3 summarizes the results of the correlation analysis of factors associated with 28-day mortality. A strong positive correlation was found between inotropic/vasopressor use and 28-day mortality (r = 0.715, p = 0.007).

Parameters affecting 28-day mortality; the presence of primary SRD flare-up, renal involvement before hospitalization, neurological involvement before hospitalization, ARF, the presence of AKI, presence of sepsis, presence of septic shock, presence of cardiogenic shock, pneumonia, need for IMV, and platelet, BUN, creatinine, ALT, PLR values were included in the logistic regression analysis. ARF, the presence of septic shock, the need for IMV, BUN level, and low PLR were significant in the final multiple-variable model using the stepwise method (forward selection). A 1-unit increase in BUN created a risk of death of 1.036; while ARF was 22.961; septic shock was 13.727; and the need for IMV was 16.893 times greater (Table 4). The PLR/100 was calculated according to the determined variable and it was found that a reduction of 100 units in the PLR increased the risk of mortality by 1.236 times.

## 4. Discussion

Prognosis has improved with immunosuppressive and immunomodulatory treatments developed in recent years as a result of a better understanding of the pathophysiology of SRDs. Despite these advancements in medicine, individuals with SRDs at any stage may require intensive care and have a high mortality rate as their outcome. Early diagnosis and treatment are very important in these diseases, which have significant morbidity and mortality. The type, etiology, clinical features, and factors affecting survival of SRDs in patients hospitalized in the ICU were investigated for these reasons. Similar to the study by Faguer et al., over a 12-year period, 1.8% of 6826 patients admitted to the ICU had a rheumatologic disease [2].

Dumas et al. investigated the outcome of 363 critically ill patients with SRDs in the most comprehensive multicenter study ever conducted [3]. Similar to the current study, the most common SRD was SLE. In other studies, however, RA was reported to be the most common SRD [4, 19]. RA can now easily be controlled due to the development of disease-modifying antirheumatic drugs (DMARDs) and new treatments, and the rate of RA-related intensive care admissions may have decreased.

SRDs are immune-related diseases that can progress with significant organ involvement. While the use of immunosuppressive and/or biological agents in current treatment approaches significantly improves the course of the disease, susceptibility to infection may increase after the use of these agents in a significant number of patients. It is critical to understand the reasons why patients with SRDs require ICU admission, because it allows intensivists and rheumatologists to provide early targeted therapy, which will reduce morbidity and mortality. Infections and flare-ups of the primary disease were the most common reasons for admission to the ICU for patients with SRDs, as expected. Given that approximately half of the patients in this study had organ involvement and a history of immunosuppressive agent use in the previous 3 months, the most common reason for ICU admission is not surprising. According to previous research, the most common conditions requiring ICU admission were infection and SRD flare-up [1, 2, 5]. According to the literature, the rate of infection, which was one of the main reasons for ICU admission, ranges from 32% to 64%, and the rate of disease flare-up ranges from 23% to 54%, both of which were supported by this current study [4]. It was also revealed in this study that a significant proportion of the ICU admissions were for SRD-unrelated reasons. Thus, it is important to remember that other causes should be taken into account in the differential diagnosis in addition to the most common ones.

During admission, the most common organ dysfunctions were respiratory (from 36.9% to 58.3%) [5, 20], cardiac (from 27.6% to 57.5%) [20, 21], and renal (from 28.7% to 66.7%) [5, 20] involvement. Neurological, hematological, and gastrointestinal system involvement can also be seen.

ARF, as seen in this study, is a significant cause of morbidity and mortality in patients with SRDs. Many structures in the respiratory system, particularly the pleura, airway, alveoli, lung parenchyma, and vascular structures in the lung, can be affected by SRDs, resulting in organ involvement [1, 17]. ARF may be the cause of the disease’s first clinical manifestation and even the reason for admission to the ICU. AAV, RA, and systemic sclerosis, particularly SLE, were the most common SRDs in the patients admitted to the ICU in the current study, and these diseases may cause significant lung involvement. Patients with lung involvement who use immunosuppressive or biological agents concurrently are more vulnerable to respiratory tract infections. Shi et al. investigated the etiology and outcome of SRD patients admitted to the ICU due to ARF [17]. SLE was reported to be the most common diagnosis, infection and flare-up of the primary disease were the most common causes of ARF, and mortality was high.

Renal involvement was found in a significant number of the patients. AKI, RRT, and BUN elevation were statistically significantly higher in the nonsurvivor group. A high BUN level was an independent risk factor for mortality. SLE and AAV can be especially associated with renal involvement [22, 23], and this patient group constituted a significant proportion in the current study. It is clear that the presence of a high rate of sepsis and septic shock contributes to organ dysfunction and causes AKI and the need for RRT in patients with or without organ involvement. Reasons such as sepsis, the presence of shock, and steroid use may also have caused high BUN levels.

Patients with SRDs may have hematological involvement and require ICU admission. Anemia, leukopenia, thrombocytopenia, lymphadenopathy, and/or splenomegaly are all common at the time of diagnosis and throughout the course of SLE. These abnormalities could be a sign of SLE, or they could be caused by other diseases or the treatment. Lymphopenia can be seen in 20% to 75% of SLE patients, especially when the disease is active [24]. Lymphopenia may be associated with an increased risk of infection or disease activation in SLE patients. The most common cause of thrombocytopenia in SLE patients is immune thrombocytopenia, which is caused by immune-mediated destruction of platelets and megakaryocytes. Other mechanisms of thrombocytopenia in SLE include platelet depletion, splenomegaly, and bone marrow suppression. Thrombocytopenia can also be seen in RA patients. In this study, the rate of death was statistically considerably greater in individuals with thrombocytopenia, while there was no statistically significant difference in mortality in patients with hematological involvement. Both groups had lymphopenia, and there was no statistically significant difference. In previous studies, it was seen that an increase in the PLR in rheumatological illnesses can be a significant indicator of inflammation [25]. Additionally, it was demonstrated that an increase in the PLR in critically ill sepsis patients can act as a crucial mortality marker [26]. In contrast to the findings of previous research, it was found herein that a decline in the PLR increased mortality. In the current study, patients with sepsis and septic shock had statistically significantly higher mortality rates. It was observed that septic shock was an independent risk factor for death. Thrombocytopenia may appear in sepsis patients as a result of direct bone marrow suppression, microcirculation disruption, or the development of organ dysfunction. In the present research, platelets may have decreased more than lymphocytes due to these factors, and the decline in the PLR may be an independent risk factor for mortality. Nevertheless, no studies that examined the PLR as a marker in indicating mortality in severely ill systemic rheumatologic patients could be found in the literature.

Previous studies have shown that shock, the use of vasopressors, the need for renal replacement, and mechanical ventilation are associated with poor prognosis [1, 19, 27, 28]. In the current study, septic shock and the need for IMV were independent risk factors for mortality.

In intensive care, the mortality rate for patients with SRDs ranges from 16% to 80% [17]. Many factors can influence mortality rates, including underlying diseases, infection, sepsis, shock, and organ involvement. The high APACHE II score, septic shock, organ involvement, and ARF rates in this study may also explain the high mortality rate.

In this paper, the extended laboratory and clinical factors associated with mortality (ICU, 28-day, and 6-month) were examined in detail, as were the reasons for admission to the ICU. The current results in critical rheumatologic patients with significant morbidity and mortality were presented. Nevertheless, there were also some limitations of the study. First, as it was retrospective and conducted at a single center, which may have affected the quality of the study. Second, rheumatic diseases are a very heterogeneous group of diseases with different clinical manifestations; thus, the findings cannot be generalized to all rheumatic diseases. Finally, due to the heterogeneity of rheumatological patients hospitalized in the ICU and the fact that immunosuppressive treatments can be given to a small number of patients, the relationship between the administration of these drugs during their follow-up in the ICU and mortality could not be evaluated statistically.

In conclusion, the most common SRD and cause of ICU admission were SLE, and the combination of infection and flare-up of the primary disease, respectively. It should be kept in mind that hospitalization may be unrelated to the disease in approximately 20% of patients. In critically ill rheumatologic patients, ARF, septic shock, the need for IMV, a high BUN level, and a low PLR might be important predictors of mortality.

## Figures and Tables

**Table 1:** Demographic and clinical characteristics of SRD patients admitted to the ICU based on 28-day mortality.

Variable	Overall n = 127	Nonsurvivors n = 78	Survivors n = 49	p-value

Age (years), mean ± SD	48.55 ± 17.05	49.77 ± 16.61	46.62 ± 17.72	0.312

Sex, n (%)				
Female	83 (65.4)	54 (69.2)	29 (59.2)	
Male	44 (34.6)	24 (30.8)	20 (40.8)	0.247

Admission to the ICU, n (%)				
From emergency service	25 (19.7)	12 (15.4)	13 (26.5)	
From inpatient service	99 (78)	64 (82.1)	35 (71.4)	0.306
From another ICU	3 (2.4)	2 (2.6)	1 (2)	

Diagnosis, n (%)				
SLE	41 (32.3)	24 (30.8)	17 (34.7)	
RA	22 (17.3)	15 (19.2)	7 (14.3)	
AAV	22 (17.3)	13 (16.7)	9 (18.4)	0.300
Systemic sclerosis	19 (15)	15 (19.2)	4 (8.2)	
Other*	23 (18.1)	11 (14.1)	12 (24.5)	

Duration of the disease (years),				
Median (Q1–Q3)	4 (1–11)	4 (1–11.5)	3(1–11)	0.608

APACHE II score, mean ± SD	24.74 ± 11.19	28.55 ± 9.95	18.65 ± 10.41	0.001

Charlson Comorbidity Index, median (Q1–Q3)	3 (1–4)	3 (2–5)	2 (1–4)	0.022

Length of stay (days), median (Q1–Q3)	6 (3–12)	6 (3–11)	6 (4–14)	0.471

Reason for admission to the ICU, n (%)				
Infection	22 (17.3)	15 (19.2)	7 (14.3)	0.473
Primary SRD flare-up	28 (22)	14 (17.9)	14 (28.6)	0.160
Combined infection + primary SRD flare-up	45 (35.4)	28 (35.9)	17 (34.7)	0.890
SRD-unrelated reasons	28 (22)	19 (24.4)	9 (18.4)	0.648
Drug side effects	5 (3.9)	4 (5.1)	1 (2)	-
SRD-related complications	1 (0.8)	-	1 (2)	-

Organ involvement of the disease, n (%)				
Respiratory	63 (49.6)	41 (52.6)	22 (42.9)	0.400
Renal	51 (40.2)	35 (44.9)	16 (32.7)	0.172
Neurological	15 (11.8)	7 (9)	8 (16.3)	0.211
Cardiovascular	13 (10.2)	9 (11.5)	4 (8.2)	0.541
Gastrointestinal	3 (2.4)	3 (3.8)	-	0.283
Hematological	16 (12.6)	9 (11.5)	7 (14.3)	0.650
Other	12 (9.4)	6 (7.7)	6 (12.2)	0.535
No organ involvement	17 (13.4)	14 (17.9)	3 (6.1)	0.057

Organ involvement leading to hospitalization in the ICU, n (%)				
Respiratory	98 (77.2)	63 (80.8)	35 (71.4)	0.222
Renal	43 (33.9)	26 (33.3)	17 (34.7)	0.993
Neurological	21 (16.5)	12 (15.4)	9 (18.4)	0.660
Cardiovascular	18 (14.2)	11 (14.1)	7 (14.3)	0.977
Gastrointestinal	4 (3.1)	4 (5.1)	-	0.159
Hematological	11 (8.7)	7 (9)	4 (8.2)	1.000
Other	1 (0.8)	1 (2)	-	-

Treatment in the last 3 months, n (%)	72 (56.7)	48 (61.5)	24 (49)	0.097
Steroid, n = 124	15 (11.8)	12 (15.4)	3 (6.1)	0.230
Disease-modifying antirheumatic drugs, n = 63				
Cyclophosphamide, n = 63	17 (13.4)	11 (14.1)	6 (12.2)	0.841
Rituximab, n = 63	6 (4.7)	3 (3.8)	3 (6.1)	0.363
Azathioprine/mycophenolate mofetil, n = 63	22 (17.3)	16 (20.5)	6 (12.2)	0.455
Antitumor necrosis factor, n = 63	5 (3.9)	2 (2.6)	3 (6.1)	0.323

Steroid dose, n (%)				
None	52 (40.9)	27 (34.6)	25 (51)	
<7.5 mg	26 (20.5)	17 (21.8)	9 (18.4)	
7.5–30 mg	11 (8.7)	6 (7.7)	5 (10.2)	
30–100 mg	19 (15)	12 (15.4)	7 (14.3)	0.291
>100 mg	16 (12.6)	13 (16.7)	3 (6.1)	
Unknown	3 (2.4)	3 (3.8)	-	

ARF, n (%)	107 (84.3)	76 (97.4)	31 (63.3)	0.001

AKI, n (%)	81 (63.8)	58 (74.4)	23 (46.9)	0.002

Pneumonia, n (%)	87 (68.5)	59 (75.6)	28 (57.1)	0.029

Sepsis, n (%)	107 (84.3)	71 (91)	36 (73.5)	0.008

Shock, n (%)	78 (61.4)	67 (85.9)	11 (22.4)	0.010

Type of shock, n (%)				
Septic	73 (57.5)	63 (80.8)	10 (20.4)	0.001
Cardiogenic	20 (15.7)	17 (21.8)	3 (6.1)	0.018
Hypovolemic	2 (1.6)	2 (2.6)	-	-

Treatments applied in the ICU, n (%)				
NIMV	56 (44.1)	35 (44.9)	21 (42.9)	0.824
IMV	85 (66.9)	71 (91)	14 (28.6)	0.001
RRT	44 (34.6)	34 (43.6)	10 (20.4)	0.008
Inotropic/vasopressor therapy	81 (63.8)	71 (91)	10 (20.4)	0.001
Plasmapheresis	37 (29.11)	23 (29.5)	14 (28.6)	0.912

ICU: Intensive care unit, SLE: systemic lupus erythematosus, RA: rheumatoid arthritis, AAV: antineutrophil cytoplasmic antibody (ANCA)-associated vasculitis, APACHE II score: acute physiologic and chronic health evaluation, SRD: systemic rheumatic disease, NIMV: non-invasive mechanical ventilation, RRT: renal replacement therapy, ARF: acute respiratory failure, AKI: acute kidney injury.

* Other: Behçet disease, dermatomyositis, polymyositis, familial Mediterranean fever, adult-onset Still’s disease, gout, ankylosing spondyloarthritis, Sjögren’s syndrome, mixed connective tissue disease, undifferentiated connective tissue disease.

**Table 2:** Laboratory parameters of patients with systemic rheumatic disease admitted to the ICU based on 28-day mortality.

Variable	Overall n = 127	Nonsurvivors n = 78	Survivors n = 49	p-value
White blood cell (10^3^/μL), median (Q1–Q3)	9.48 (6.3–15.7)	9.1 (5.58–17.89)	9.6 (7.65–14.95)	0.586
Neutrophil (10^3^/), median (Q1–Q3)	7.65 (5.18–13.27)	7.465 (4.37–14.4)	8 (5.9–11.72)	0.656
Lymphocyte (10^3^/μL), median (Q1–Q3)	0.800 (0.400–1.520)	0.800 (0.400–1.497)	0.910 (0.400–1.720)	0.594
Monocyte (10^3^/μL), median (Q1–Q3)	0.500 (0.200–0.930)	0.500 (0.200–0.900)	0.470 (0.250–0.940)	0.647
Hemoglobin (g/dL), median (Q1–Q3)	9.80 (8–11.7)	9.85 (8.1–11.9)	9.5 (7.65–11.5)	0.502
Platelet (10^3^/μL), median (Q1–Q3)	185 (102–316)	144 (74.5–273.75)	221 (138–361)	0.010
Mean platelet volume (fL), mean ± SD	9.06 ± 1.61	9.17 ± 1.67	8.89 ± 1.52	0.370
Platelet crit, median (Q1–Q3)	0.16 (0.09–0.28)	0.15 (0.084–0.305)	0.213 (0.121–0.178)	0.135
CRP (mg/L), median (Q1–Q3)	77.95 (23–142.25)	74.4 (25.83–150.75)	80.4 (20.48–135.5)	0.948
CRPmax (mg/L), median (Q1–Q3)	134.5 (70.08–242)	109.5 (55.93–223)	148 (81.8–266.25)	0.355
Procalcitonin (ng/mL), median (Q1–Q3)	0.73 (0.23–3.5)	1.13 (0.28–6.18)	0.48 (0.1–2.87)	0.111
Procalcitonin maximum (ng/mL), median (Q1–Q3)	2.94 (0.73–16.33)	5.45 (1.36–23.5)	1.7 (0.31–9)	0.005
BUN (mg/dL), median (Q1–Q3)	37.5 (21–66)	40.87 (24.75–71.1)	26.7 (10.61–53.81)	0.037
Creatinine (mg/dL), median (Q1–Q3)	1.26 (0.76–2.77)	1.39 (0.92–3.11)	0.98 (0.65–2.42)	0.114
Glomerular filtration rate <60, n (%)	57 (44.9)	37 (47.4)	20 (40.8)	0.465
Serum albumin (g/L), mean ± SD	2.44 ± 0.64	2.33 ± 0.65	2.6 ± 0.59	0.020
Alanine aminotransferase (U/L), median (Q1–Q3)	21 (14–45)	23.5 (15.75–61.25)	19 (12.5–37)	0.096
Lactate (mmol/L), median (Q1–Q3)	2.15 (1.39–3.5)	2.8 (1.73–5.78)	1.65 (0.98–2.35)	0.001
Ferritin (mg/dL), median (Q1–Q3)	502.5 (130.3–1499)	804 (140.7–3110)	425.6 (130–884)	0.156
CRP/albumin ratio, median (Q1–Q3)	31.88 (9.09–65.13)	33.36 (10.2–65.13)	28.95 (7.6–67.05)	0.674
NLR, median (Q1–Q3)	10.14 (5.38–19.75)	10.24 (4.65)	9.5 (5.77–19.18)	0.937
PLR, median (Q1–Q3)	230 (116.84–370)	233.33 (93.85–327.64)	223.33 (155.96–484.21)	0.137
Lymphocyte to monocyte ratio, median (Q1–Q3)	1.81 (0.83–3)	1.61 (0.8–3)	2.15 (0.84–3)	0.651

CRP: C-reactive protein, BUN: blood urea nitrogen, GFR: glomerular filtration rate, NLR: neutrophil-lymphocyte ratio, PLR: platelet-lymphocyte ratio.

**Table 3:** Results of the correlation analysis of factors associated with 28-day mortality.

Variable	r-value	p-value

APACHE II score	0.432	<0.001

Charlson comorbidity index	0.204	0.022

Steroid dose	0.168	0.062

ARF	0.457	<0.001

Shock	0.634	<0.001

Sepsis	0.235	0.008

Septic shock	0.594	<0.001

Acute kidney failure	0.278	0.002

Treatments applied in the ICU		
IMV	0.646	<0.001
RRT	0.237	<0.001
Inotropic/vasopressor therapy	0.715	0.007

Platelet	−0.230	0.009

Maximum procalcitonin	0.259	0.004

BUN	0.186	0.037

Serum albumin	−0.210	0.02

Lactate	0.408	<0.001

**Table 4:** Logistic regression for the independent risk factors for 28-day mortality.

	Odds ratio (95% Cl)	p-value
ARF	22.961 (2.545–207.149)	0.005
Septic shock	13.727 (3.632–51.879)	<0.001
IMV	16.893 (3.543–80.548)	<0.001
BUN	1.036 (1.012–1.061)	0.004
PLR (for each 100-unit decrease)	1.236 (1.037–1.473)	0.018

CI: Confidence interval.
